# Supplementary material for: Cusp bifurcation in a metastatic regulatory network
Source: J Theor Biol. Author manuscript; Available in PMC 2024 May 28. (PMC11132523; doi:10.1016/j.jtbi.2023.111630)
Supplement: Supplementary Material [file NIHMS1995597-supplement-Supplementary_Material.pdf]

Cusp bifurcation in a metastatic regulatory network : Supplementary Materials

Brenda Delamonica[1], Gábor Balázsi[*2], and Michael Shub[**3]

[1]Applied Mathematics and Statistics Department, Stony Brook University, Stony Brook, NY 11794, USA

[2]The Louis and Beatrice Laufer Center for Physical and Quantitative Biology, Stony Brook University, Stony Brook, NY 11794, USA , Department of Biomedical Engineering Department, Stony Brook University, Stony Brook, NY 11794, USA

[3]Department of Mathematics,City College and the Graduate Center of CUNY

$*, **$ Corresponding authors: gabor.balazsi@stonybrook.edu, shub.michael@gmail.com

Declaration of Interest: None.

1 Appendix A

Here we present some elementary examples of biological systems that exhibit cusp bifurcations. We include an extensive analysis of the mathematics involved in solving such systems for those who are interested.

1.1 Cusp Bifurcation in biological networks

1.1.1 Gene circuits

The cusp point is relevant to many areas of biology besides metastasis. It should be found for gene networks described earlier as bistable or multistable. This applies to the toggle switch, one of the foundational gene circuits in synthetic biology. Thus, we considered the 2-gene and a 3-gene networks from Appendix 1A and 1C of Smale and Rajapakse [10], as other examples of a cusp bifurcation in biological systems. Smale and Rajapakse showed that pitchfork bifurcations exist for these networks. Below we give an analysis of the 2-gene network.

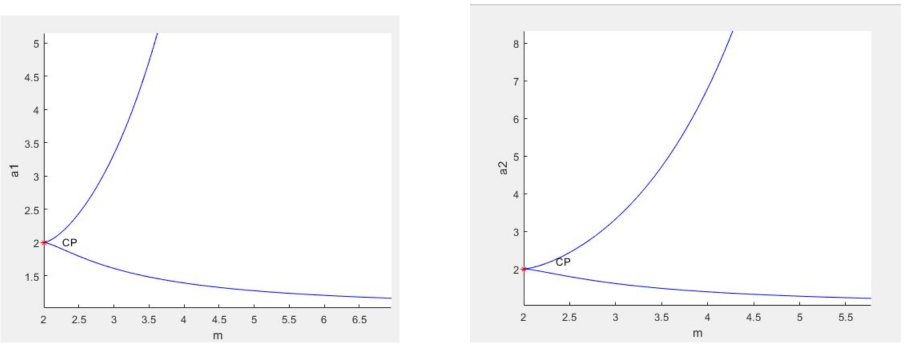

(a) Plot varying α_1, m where $\alpha_2 = 2$

(b) Plot varying α_2, m where $\alpha_1 = 2$

Figure 1: MATCONT 2-gene cusp results

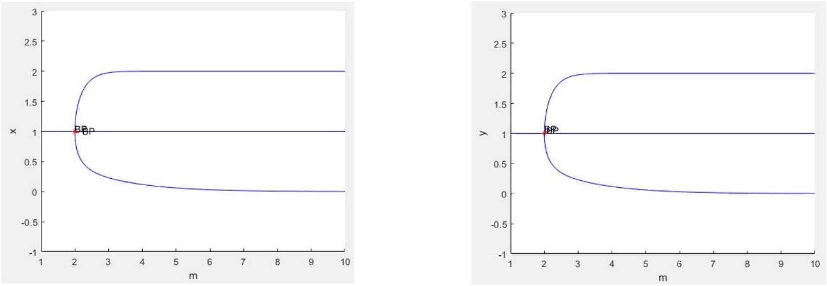

(a) Plot of x, m (b) Plot of y, m

Figure 2: MATCONT pitchfork bifurcation

We used MATCONT to show that a cusp point occurs for the system

$$\dot{x} = \frac{\alpha_1}{1 + y^m} - x$$
$$\dot{y} = \frac{\alpha_2}{1 + x^k} - y$$

In Figure 1a we set $\alpha_2 = 2$ and $m = k > 0$. As we varied α_1 and m we found a cusp exists at $(x, y, \alpha_1, m) = (1.00, 1.00, 2.00, 1.99)$. In Figure 1b we set $\alpha_1 = 2$, $m = k > 0$ and varied α_1 and m. A cusp point exists at $(x, y, \alpha_2, m) = (1.00, 1.00, 2.00, 1.99)$. This aligns with Smale and Rajapakse's results [10]. They set $\alpha_1 = \alpha_2 = 2$, $m = k > 0$ and proved that a pitchfork bifurcation occurs at the point $m = 2$. They note that for all $0 \leq m < 2$ there is only one solution to the one parameter system which is $(x, y) = (1, 1)$. It can be shown that for values past the cusp, or when $m > 2$, there are three solutions and we have the same pitchfork scenario as we described in the paper. We plot the pitchfork using MATCONT in Figure 2.

In Figure 3 we used the same fimiplicit3 plot in MATLAB as we did in the paper. We plot the solution surface of x and y separately and vary the two relevant parameters (α_1, m) and (α_2, m) respectively. The surfaces both are folds which we would expect to see for a cusp bifurcation. Figure 1 shows the projection of the fold on the (α_1, m) and (α_2, m) plane. As for the

metastasis-regulatory network, the cusp point separates the parameter ranges for continuous versus discontinuous transitions.

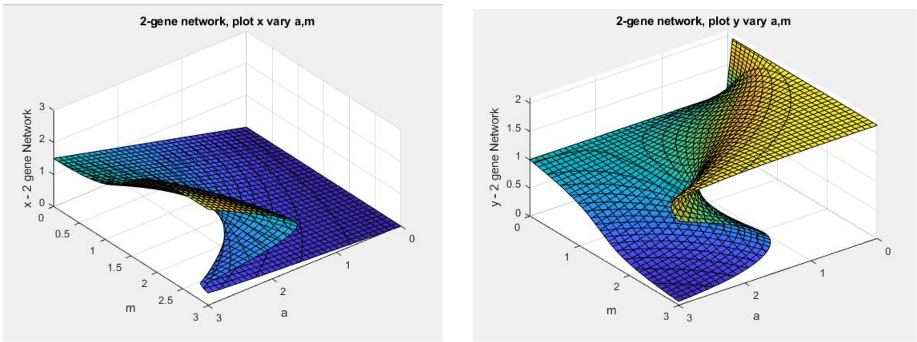

(a) 2-gene x, α_1, m plot

(b) 2-gene y, α_2, m plot

Figure 3: Matlab fimplicit3 plots

1.1.2 Cell Division

Another important network for which our analysis applies drives the cell cycle. Rajapakse and Smale [11] suggest that the pitchfork bifurcation may explain some of the properties of cellular division. The decision of cells between cycling and senescence is driven by a bistable network, the core of which is a variant of the toggle switch involving CDK2 and Rb (Figure 2.A in [2]). Considering the above analysis for the toggle switch, the cell cycle network might also be modelled as a Toggle, with a pitchfork or cusp bifurcation. Thus, we use the 2-gene Toggle network from Smale and Rajapakse [10] to demonstrate what could be happening during cell division and how it relates to a cusp bifurcation. We use the toggle switch to illustrate some biologically relevant features of the dynamics and note that the bifurcations of the cell cycle network will occur at different, currently unknown parameter values.

Since the toggle switch has two steady states, one of which corresponds to entering the cell cycle, we need to assign a biological meaning to the other steady state. We propose that the other

steady state corresponds to differentiation, which is known to be antagonistic with cell cycling [7] [6].

The dynamics of a differential equation with fixed parameters after the pitchfork or cusp bifurcations are:

1. There are 3 equilibria, two sinks and a saddle.

2. The forward orbit of any point tends to one of the equilibria.

3. The orbits which tend to the saddle, called the stable manifold of the saddle, form an (n-1) dimensional disc which separates the two basins of the stable equilibria.

In Figure 4 we produce a vector plot using the 2-gene Toggle network from Smale and Rajapakse [10]. We see all three behaviors listed in this image, where the upper sink could be low CDK2/high Rb, the lower sink is high CDK2/low Rb and the $x = y$ line divides paths a cell may take as they differentiate if they initiate above the line or proliferate if they begin on a path below the line.

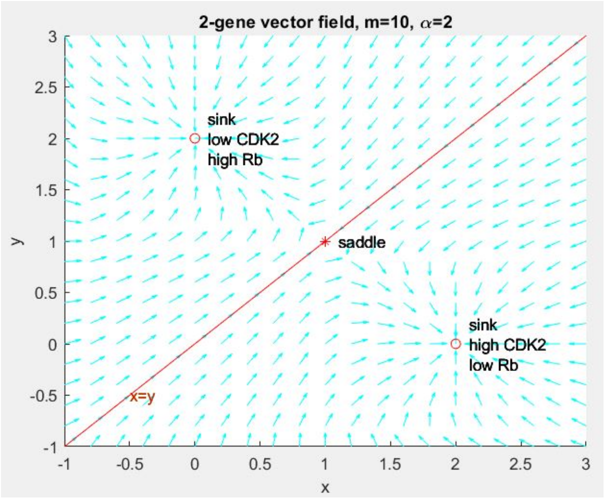

Figure 4: Vector plot of 2 gene circuit system to show 3 equilibria [10]

Almost all orbits tend to one of the stable equilibria. An orbit starting near the red line will have very slow dynamics. Paths close to the unstable equilibria and far from the stable equilibria will take a long time to arrive near a stable equilibrium. In the case of cell division one stable equilibrium corresponds to the cell being proliferative, and the other to cell differentiation. Points close to the stable manifold will have fast dynamics as they tend to the sink. However, points close to the unstable equilibria and far from the stable equilibria will define cells that may appear quiescent for a long time, although ultimately they will commit to cycling. Cells distributed along the green arrows could have drastically long waiting times before entering the cell cycle, as observed in the experiments.

We see the same behavior in 3 dimensions. The 3 dimensional system is defined by Smale and Rajapakse as [10] follows:

$$\dot{x} = \frac{\alpha}{1+z^m} - x$$
$$\dot{y} = \frac{\alpha x^m}{1+x^m} - y$$
$$\dot{z} = \frac{\alpha}{1+y^m} - z$$

To find the behavior in the bistable region we set $\alpha = 2$ and $m = 3$. In Figure 5 we show the 3 equilibria as black stars, and the forward orbits are the blue dotted lines each tending towards one of the equilibria. The eigenspace of the saddle is a plane that divides two regions in three dimensions, separating the two basins of stable equilibria. This plane is an approximation of the stable manifold of the saddle. We use `ode45` in MATLAB to solve the system starting from various points in the space. Most points stay on the same side of the manifold as where they started.

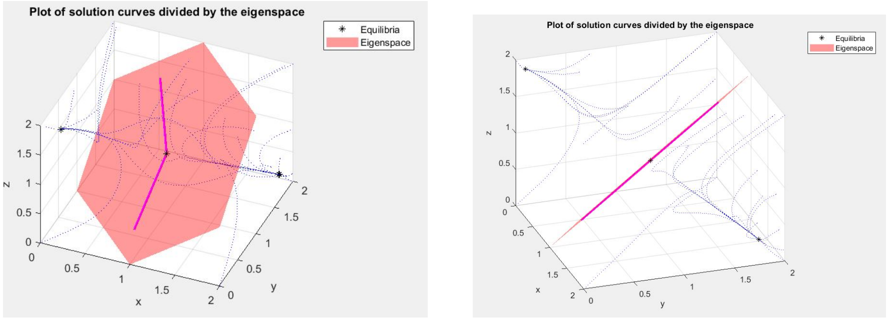

(a) (b)

Figure 5: Matlab plot of eigenspace and solution curves in blue. Lines in magenta are the eigenvectors of the saddle point which determine the eigenspace.

We suggest that the same behavior is seen in the system involving CDK2, Rb and Ef2 (Figure 2.A in [2]). In our model we may interpret x, y, z as EF2, CDK2, Rb respectively. In this space as z increases x, y decrease and solutions tend to the stable sink in the upper left hand corner of Figure 5b, which corresponds to differentiation. Conversely, as z decreases, x, y increase and solutions tend to the sink in the lower right hand corner of Figure 5b, which corresponds to cell cycle entry. This reflects a switch behavior in cell division with 3 variables.

There is a symmetry in the 2-gene model when $m > 2, \alpha_1 = \alpha_2$. In two dimensions the line $x = y$ is the stable manifold that separates the two cell behaviors. Any initial point with $x > y$ or $x < y$ will converge to the same stable equilibirum. Similarly when considering the 3 dimensional model the stable manifold of the saddle separates the space such that all orbits tend to the sink of the same side. While our model is the linear approximation of the stable manifold it is clear that for values of x that are large enough, all orbits tend to the stable state which corresponds to high CDK2 concentrations. This is the observed situation with CDK2 and proliferation [2]. In the examples the line in 2-dimension and the disc or eigenspace in 3-dimension approximate the true manifold and are

based off simple gene networks, however, it could be interesting to develop a more accurate model of cell division to combine the findings in the two papers referenced [10, 2]. Furthermore, one could demonstrate that a pitchfork or cusp bifurcation exists in cell division as well.

2 Appendix B: Cusp Bifurcation Methods

So far our analysis has been based on the rather fine numerical work of Lee et. al.[1] represented in Figure 1 of the paper. But we may give a more analytical treatment of the figures which in this case and much more generally establish the existence of the cusp point and give a sharper description of the geometry of the division into monostable and multistable regions near it. The discussion of how the stable states change along paths in the parameter space will be the same in this greater generality. The subject matter is called bifurcation theory. We are given smooth differential equations $\dot{x} = V(x, \alpha)$ as above where $x \in \mathbb{R}^n$ and α is a parameter in $\mathbb{R}^j$ and $V : \mathbb{R}^n \times \mathbb{R}^j \to \mathbb{R}^n$. In our case α is a real parameter so $j = 1$.

Bifurcation theory studies how the behavior of the solution of V changes as α changes. This is a very well developed subject [4, 8, 3]. The two stable bifurcations which will interest us are the saddle-node and cusp bifurcations. We will use the referenced numerical methods to find them. [9, 5] The equation 1) $V(x, \alpha) = 0$ defines the equilibria. Recall that in general

$$V(x, \alpha) : \mathbb{R}^n \times \mathbb{R} \to \mathbb{R}^n$$

and we assume that when $V(x, \alpha) = 0$

equation 2) $DV(x, \alpha)$ goes from $\mathbb{R}^{n+1} \to \mathbb{R}^n$ and the rank of the derivative is n.

So the set of zeros is locally a curve near (x, α) by the implicit function theorem.

We assume one of the eigenvalues of the derivative $D_x V(x, \alpha)$ is 0, so we add

equation 3) $Det[D_x V(x, \alpha)] = 0$

and now we assume that

$$(V(x, \alpha), Det[D_x V(x, \alpha)]) : \mathbb{R}^n \times \mathbb{R} \rightarrow \mathbb{R}^n \times \mathbb{R}$$

has rank $n + 1$.

So x, α is an isolated solution of equations 2) and 3), and the curve of zeros is tangent to the α =constant plane. A saddle of index k and one of index $k - 1$ collide and annihilate or appear if the parameter runs in the opposite direction.

Here is an illustration of the saddle node bifurcation from Scholarpedia in one dimension and one parameter.

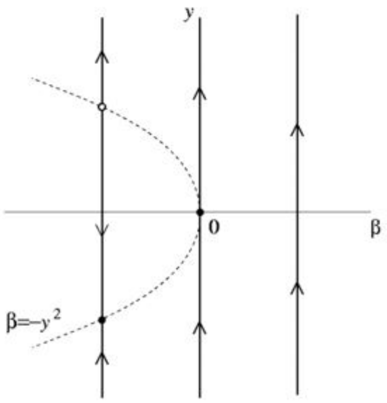

Figure 6: Saddle-node bifurcation in the one-dimensional system [9]

If $n = 2$ or higher, here is the dynamical picture in the plane. In general the downward arrow represents an $(n - 1)$ dimensional plane of the non-zero eigenvalues.

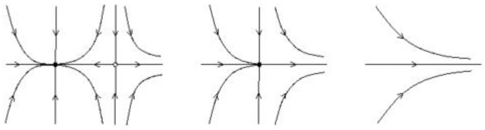

Figure 7: Saddle-node bifurcation on the plane in the system [9]

Now if we have two parameters ρ and k

$$V(x, \rho, k) : \mathbb{R}^n \times \mathbb{R}^2 \to \mathbb{R}^n$$

Then

$1')V(x, \rho, k) = 0$

defines the set of equilibria and we assume

$2')$the rank of $D(V(x, \rho, k)) = n$

so the set of equilibria near (x, ρ, k) is a 2-dimensional surface. On the surface we have those equilibria with a zero eigenvalue where the number of equilibria might be changing, these are described by adding the equation

$3')$ $Det[D_x V(x, \rho, k)] = 0$

and we assume that the map $(x, \rho, k) \to (V(x, \rho, k), Det[D_x V(x, \rho, k)])$ has rank $n + 1$ where the two equations are satisfied. Hence the solution is a curve near (x, ρ, k).

When we project the curve on the parameter plane, (ρ, k) there is either A) no singularity of the projection, i.e. no tangent vector to the curve is vertical or B) there is a singularity of the projection, that is a tangent vector to the curve which is vertical.

In case A) there are two possibilities:

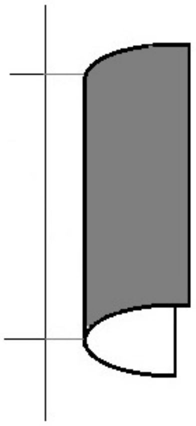

Figure 8: Fold projected on parameter plane

Aa) 0 is a simple eigenvalue of $D_x(V(x, \rho, k)$ along the curve. In this case the projected curve divides the plane near (ρ, k). On one side there is no equilibrium on the other side, there are two of index j and $j-1$. So in a region of interest to us, a saddle and a sink. If a curve in the parameter plane crosses the projected curve we see a saddle-node bifurcation.

This is called a fold bifurcation.

Ab)There is a point along the curve where the rank of $D_xV(x, \rho, k)$ is $n-1$ but a second eigenvalue is 0. Now when a curve crosses the projected curve we may create a saddle of index j and another equilibria of index $j-1$ or $j+1$ depending on where the crossing takes place and for some values of the parameter there are limit cycles. We don't see this behavior in the data of Lee et al. and we will not discuss it. This bifurcation is called a Bogdanov-Takens bifurcation.

There are generically five types of bifurcations of equilibria depending on two parameters (See Kuznetsov). All might be interesting for biology.

Case B) is the one that interests us. It is the cusp bifurcation. There is a singularity of the projection, that is a tangent vector to the curve which is vertical. Recall that the tangent to the

curve is the null space of the derivative of $(V(x,\rho,k), Det[D_xV(x,\rho,k)])$. Now for a vertical vector $(v,0,0)$ to be in the null space it is necessary and sufficient that

4′) $D_x(V(x,\rho,k)(v) = 0$ and

5′) $\nabla_x Det[D_xV(x,\rho,k)] \bullet (v) = 0$

The references above give methods for determining that these equations have a solution. When equations 1′) and 3′) are satisfied $D_xV(x,\rho,k)$ has a kernel as $Det[D_xV(x,\rho,k)] = 0$ so we would like to express the vector v in terms of (x,ρ,k). Assuming that $D_xV(x,\rho,k)$ has rank $n-1$ when $V(x,\rho,k) = 0$ then at least one of the $(n-1)$ by $(n-1)$ minors has non-vanishing determinant and the adjugate matrix is not zero. By Cramer's Rule any non-zero column of the adjugate matrix is in the kernel of $D_xV(x,\rho,k)$.

So a cusp point is defined as a non-degenerate solution of the $n+2$ by $n+2$ system of equations

$$\begin{aligned} V(x,\rho,k) &= 0 \\ Det[D_xV(x,\rho,k)] &= 0 \\ \nabla_x Det[D_xV(x,\rho,k)] \bullet (v) &= 0 \end{aligned} \tag{1}$$

Where v is a non-zero column of the adjugate matrix of $D_xV(x,\rho,k)$.

Here are some pictures of the cusp bifurcation when $n = 1$.

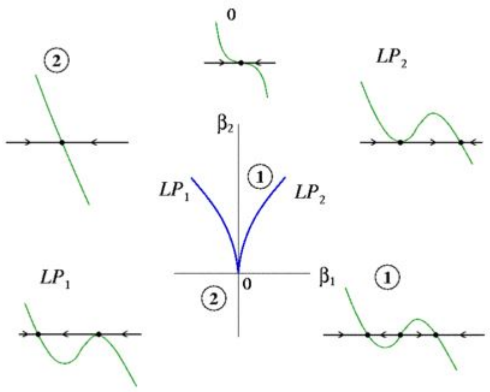

Figure 9: Cusp bifurcation in the one-dimensional system [5]

The surface below is the surface of equilibria. Outside of the cusp region there is one equilibrium and inside 3.

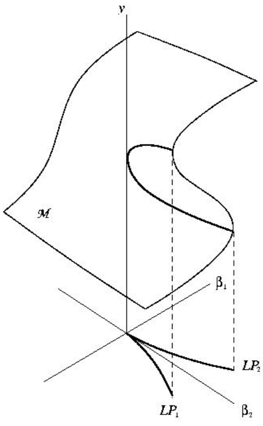

Figure 10: Equilibrium manifold near a cusp bifurcation [5]

As a path crosses the cusp curve away from the cusp point itself the equation has a saddle-node bifurcation. The picture of cubics illustrates again what is happening in 1-dimension. The two curves of the cusp are tangent at the cusp point. So the cusp point is a place where the hysteresis effect may in some sense be briefest. See questions 1 and 2 above.

A strategy for the analysis of equilibria might be to look for non - degenerate solutions of $V(x,\alpha)=0$ and $Det[D_xV(x,\alpha)]=0$ for saddle node bifurcations and of the system of equations (2) where $D_xV(x,\alpha)(v)=0$, $Det[D_xV(x,\alpha)]\bullet(v)=0$ and v is a column of the adjugate matrix of $D_xV(X,\alpha)$ for cusp bifurcations. The region in Figure 1 looks like there might be a cusp bifurcation which puts a sharper definition of the regions and supports the cell transitions in Figure 2 of the paper.

3 Appendix C: Matlab Code

Figure 1

```
%function zB = funB(t,B)

function zB = funB(B)

%no shBACH1 here


global s S c K m b r rho a


zB = s+(S-s)*K^b/(K^b+B^b) - B*( 1+ (a/(m^r*rho^r*(1+B)^r+1)) * (c/(1+c*B)) );


function [Ld,Lt] = LOSSderiv(B)


global s S c K m b r rho a


% loss term

Lt = B.*(1+ (a./(m^r*rho^r*(1+B).^r+1)).*(c./(1+c*B)));
```

```
fderiv_num1=(m^r*rho^r*(1+B).^r+1).^2.*(1+c*B).^2;

fderiv_num2=a*c*(m^r*rho^r*(1+B).^r+1).*(1+c*B);

fderiv_num3=a*c^2*B.*(m^r*rho^r*(1+B).^r+1);

fderiv_num4=a*c*m^r*rho^r*r*B.*(1+B).^(r-1).*(1+c*B);

fderiv_den=(m^r*rho^r*(1+B).^r+1).^2.*(1+c*B).^2;


% fd1=1+a*c./((m^r*rho^r*(1+B).^r+1).*(1+c*B));

% fd2=B.*(a*c^2*(m^r*rho^r*(1+B).^r+1))./((m^r*rho^r*(1+B).^r+1).^2.*(1+c*B).^2);

% fd3=B.*(a*c*m^r*rho^r*r*(1+B).^(r-1).*(1+c*B))./((m^r*rho^r*(1+B).^r+1).^2.*(1+c*B).^2);



% derivative of loss term

Ld=(fderiv_num1+fderiv_num2-fderiv_num3-fderiv_num4)./fderiv_den;

%Ld=fd1-fd2-fd3;


clear all; close all;


global s S c K m b r rho a alpha


L=3;

paramrange=logspace(-L,L,100);


B=logspace(-5,3,100);
```

```
colorsetR=[0.5 0 0; 1 0 0; 1 .5 .8];

colorsetB=[0 0 0.5; 0 0 1; 0 1 1];


clear isbist;clear lowBACH1;

s=0.02;

S=20;

c=200;

m=2;

b=3;

r=5;

a=1000;

% scan rho: RKIP degradation rate

% scan alpha: shBACH1 effect

Krange=paramrange;

rhorange=paramrange;

ctr1=1;

%figure;

for rho=rhorange

    ctr2=1;

    for K=Krange

        lhs=B.*(1+ (a./(m^r*rho^r*(1+B).^r+1)).*(c./(1+c*B)));

        rhs=s+(S-s)*K^b./(K^b+B.^b);

        %subplot(7,7,(ctr1-1)*7+ctr2);plot(B,lhs,'LineWidth',2);hold on;plot(B,rhs,'r','LineWidth'
```

```
[Ld,Lt]=LOSSderiv(B);   % get the derivative of the loss term, and the loss term


seekdownslope=1-sign(Ld);   %TRUE is loss term's derivative is negative or 0

midP=mean(B(find(seekdownslope)));

%[a K any(seekdownslope) 3]


%chance for bistable region next

if(any(seekdownslope) && Ld(floor(mean(find(seekdownslope))))*Ld(end)<0 && Ld(floor(mean(f


    zD1=fzero('LOSSderiv',[B(1) midP]);

    zD2=fzero('LOSSderiv',[midP B(end)]);


    [Ld1,Lt1]=LOSSderiv(zD1);

    [Ld2,Lt2]=LOSSderiv(zD2);

    Gt1=(s+(S-s)*K^b./(K^b+zD1.^b));    % gain term 1

    Gt2=(s+(S-s)*K^b./(K^b+zD2.^b));    % gain term 2

    isbist(ctr1,ctr2)=(Lt1-Gt1>0)*(Gt2-Lt2>0)*(zD2-zD1>0);

    lowBACH1(ctr1,ctr2)=(Gt2-Lt2<0);

    %[a K (Lt1-Gt1) (Gt2-Lt2)]

    %sBACH1a(ctr1,ctr2)=Gt1-Lt1;  % gain term - loss term = dB/dt

    %sBACH1b(ctr1,ctr2)=Gt2-Lt2;  % gain term - loss term = dB/dt

%monostability is certain

else
```

```
            isbist(ctr1,ctr2)=0;
            zD=fzero('funB',[0 B(end)*100]);
            lowBACH1(ctr1,ctr2)=(zD<0.2);
            %sBACH1(ctr1,ctr2)=zD<0.2;
        end;
        ctr2=ctr2+1;
    end;
    ctr1=ctr1+1;
end;

xvec=log10(rhorange);yvec=log10(Krange(1:47));
[xx,yy]=ndgrid(xvec,yvec);
% figure;mesh(xvec,yvec,atan2(yy,xx));
atval=atan2(yy,xx);
atval(48,length(Krange))=0;

% figure;imagesc(log10(rhorange),log10(Krange),atval/min(min(atval)));colorbar;
% figure;imagesc(log10(rhorange),log10(Krange),isbist);colorbar;

B = bwboundaries(isbist);
%mask=isbist>0.5;

bistsurf=isbist*0.5+lowBACH1;
%bistsurf=lowBACH1;
```

```
%bistsurf(:,1:47)=(atval(:,1:47)/min(min(atval))).^0.75;

bistsurf(:,1:47)=(atval(:,1:47)/min(min(atval)));


figure;

I=imagesc(log10(rhorange),log10(Krange),bistsurf);

patch(log10(Krange(B{:}(:,2))),log10(rhorange(B{:}(:,1))),[0.8 0.7 0.2])

%imagesc(log10(rhorange),log10(Krange),sBACH1);colorbar;

hold on;cb=colorbar;

%patch(log10(rhorange(isbist)),log10(Krange(isbist)),'g');


% plot(log10(10),log10(.05),'o','Color',colorsetB(1,:),'LineWidth',2,'MarkerSize',20);

% plot(log10(10),log10(.5),'o','Color',colorsetB(2,:),'LineWidth',2,'MarkerSize',20);

% plot(log10(10),log10(5),'o','Color',colorsetB(3,:),'LineWidth',2,'MarkerSize',20);

%

% plot(log10(10),log10(.5),'^','Color',colorsetR(3,:),'LineWidth',2,'MarkerSize',16);

% plot(log10(.1),log10(.5),'^','Color',colorsetR(1,:),'LineWidth',2,'MarkerSize',16);

% plot(log10(.1),log10(5),'^','Color',colorsetB(3,:),'LineWidth',2,'MarkerSize',16);


plot(log10((Krange(47)+Krange(48))/2),log10((rhorange(50)+rhorange(51))/2),'*','Color','r','LineWi

[(Krange(47)+Krange(48))/2 (rhorange(50)+rhorange(51))/2]


set(gca,'FontSize',16,'YDir','normal');

xlabel('log_{10}(K): BACH1 insensitivity to self-repression','FontSize',16);

ylabel('log_{10}(\rho): RKIP instability','FontSize',16);
```

```
colormap(gray);grid on;caxis([0 1]);
text(0,2,{'Monostable','Pro-metastatic'},'Color',[1 1 1],'FontSize',18,'FontWeight','Bold');
text(0.1,-2.2,{'Monostable','Anti-metastatic'},'Color',[0 0 0],'FontSize',18,'FontWeight','Bold');
text(1.5,-.5,{'Bistable','Mixed'},'Color',[0 0 0],'FontSize',18,'FontWeight','Bold');
set(cb,'TickLabels',flipud(get(cb,'TickLabels')))
```

Figure 3

```
function [lastDW,lastN,N,Wx,DWd,DWw]=NewtonsMethodfcn(eqns,x0,vars,count)


NTable=[];
WxTable=[];
vars0=x0;
W=double(subs(eqns,vars,vars0));
DW=jacobian(eqns,vars);
DWsub=double(subs(DW,vars,vars0));


if rank(DW)==size(DW,2)
    DWdagger=inv(DWsub);
else
    DWdagger=DWsub'*inv(DWsub*DWsub');
end
DWW=DWdagger*W;
Nprime=vars0'-DWW;
WNprime=subs(eqns,vars,Nprime');
```

```
NTable=[vars0',Nprime];

WxTable=[W,WNprime];

for j=1:count

    W=double(WNprime);

    DWsub=double(subs(DW,vars,Nprime'));

    if rank(DW)==size(DW)

        DWdagger=inv(DWsub);

    else

        DWdagger=DWsub'*inv(DWsub*DWsub');

    end

    DWW=DWdagger*W;

    Nprime=Nprime-DWW;

    NTable=[NTable,Nprime];

    WNprime=double(subs(eqns,vars,Nprime'));

    WxTable=[WxTable,WNprime];

end

lastDW=DWsub;

lastN=Nprime;

N = double(NTable);

Wx = double(WxTable);

DWd=DWdagger;

DWw=DWW;


%script to generate table using Newtons Method function
```

```
s=.02;

S=20;

c=200;

m=2;

b=3;

r=5;

a=1000;


syms R L B rho K


assume([R L B], 'Real')

assume([R L B], 'Positive')


eqn1=(1/(1+B))-rho*R;

eqn2=((a*R^r)/(m^r+R^r))-L-c*L*B;

eqn3=s+(((S-s)*K^b)/(K^b+B^b))-B-c*L*B;


%a11*(a22*a33 - a23*a32) + a13*a21*a32 from Jacobian matrix

eqn4=(-rho)*((-B*c-1)*(-L*c-(B^(b-1)*K^b*b*(S-s))/(B^b+K^b)^2-1)

    -(-L*c)*(-B*c))+(-1/(B+1)^2)*((R^(r-1)*a*r)/(R^r+m^r)-(R^r*R^(r-1)

    *a*r)/(R^r+m^r)^2)*(-B*c);

eqns=[eqn1;eqn2;eqn3;eqn4];

vars=[R,L,B,rho,K];
```

```
%table format
SizeW=[4 6];
varTypesW={'double','double','double','double','double','double'};
varsW={'W(X0)','W(X1)','W(X2)','W(X3)','W(X4)','W(X5)'};
RowsW={'eqR','eqL','eqB','eqDet'};

vars1=[0.932146,2.218409,0.043501,1.028071,0.134353]; %cusppt from matcont
[newDW,newN,N,Wx,DWd,DWw]=NewtonsMethodfcn(eqns,vars1,vars,5);
insertT=Wx(:,1:6);
WX=table('Size',SizeW,'VariableTypes',varTypesW,'VariableNames',
    varsW,'RowNames',RowsW);
WX(:,:)=array2table(insertT)
```

Figure 4

```
s=.02;
S=20;
c=200;
m=2;
b=3;
r=5;
a=1000;
syms R L B rho K
assume([R L B], 'Real')
assume([R L B], 'Positive')
```

```
eqn1=(1/(1+B))-rho*R;

eqn2=((a*R^r)/(m^r+R^r))-L-c*L*B;

eqn3=s+(((S-s)*K^b)/(K^b+B^b))-B-c*L*B;

eqn4=(-rho)*((-B*c-1)*(-L*c-(B^(b-1)*K^b*b*(S-s))/(B^b+K^b)^2-1)
    -(-L*c)*(-B*c))+(-1/(B+1)^2)*((R^(r-1)*a*r)/(R^r+m^r)-(R^r*R^(r-
    1)*a*r)/(R^r + m^r)^2)*(-B*c);

eqns=[eqn1;eqn2;eqn3;eqn4];

vars=[R,L,B,rho,K];

rhoKFW=[]; %list to save curve coordinates in forward direction

rhoKBW=[]; %list to save curve coordinates in backward direction

vars1=[0.932146,2.218409,0.043501,1.028071,0.134353]; %cusppt from matcont

[newDW,newN,N,Wx,DWd,DWw]=NewtonsMethodfcn(eqns,vars1,vars,5);

rhoKFW=[N(4:5,end)];

rhoKBW=[N(4:5,end)];


%adjust index range to plot more points

for k=1:5

        deg=10^(-k);

        ker=null(newDW)*(deg);

        null(newDW);

        x0p=newN+ker;

        x0m=newN-ker;

        x1p=x0p;

        x1m=x0m;
```

```
        for j=1:20
            [newDW2,newN2,N2,Wx2,DWd2,DWw2]=
                NewtonsMethodfcn(eqns,x1p',vars,1);
            [newDW3,newN3,N3,Wx3,DWd3,DWw3]=
                NewtonsMethodfcn(eqns,x1m',vars,1);

            ker2=null(newDW2)*deg;
            x1p=newN2+ker2;
            rhoKFW=[rhoKFW,N2(4:5,end)];

            ker3=null(newDW3)*deg;
            x1m=newN3-ker3;
            rhoKBW=[rhoKBW,N3(4:5,end)];

        end
 end

%plot(rhoKFW(2,:),rhoKFW(1,:),'o',rhoKBW(2,:),rhoKBW(1,:),'o',0.134353,1.028071,'d')
plot(rhoKFW(2,1:11),rhoKFW(1,1:11),'-', rhoKBW(2,1:11), rhoKBW(1,1:11), '-', 0.134353,1.028071, 'd
xlabel('K')
ylabel('\rho')
```

Figure 5

```
s=.02;
```

```
S=20;
c=200;
m=2;
b=3;
r=5;
p=10;
a=1000;
syms R L B rho K
assume([R L B], 'Real')
assume([R L B], 'Positive')
eqn1=(1/(1+B))-rho*R;
eqn2=((a*R^r)/(m^r+R^r))-L-c*L*B;
eqn3=s+(((S-s)*K^b)/(K^b+B^b))-B-c*L*B;
%rho,K values from MATCONT CONTINUATION
rho0=1.028071;
K0=0.134353;
D=jacobian([eqn1;eqn2;eqn3],[R,L,B,rho,K]);
vars=[R,L,B,rho,K];

%cusp pt from MATCONT
varscusp=[0.932146,2.218409,0.043501,1.028071,0.134353];

subs(D,vars,varscusp);
null(subs(D,vars,varscusp));
```

```
nullsp=null(double(subs(D,vars,varscusp)));
nullsp(4,1);
nullsp(5,1);

vector=[nullsp(4,1)/nullsp(5,1);1];

tvec=linspace(-.01,.01,100);
Bvec=[];

for i=1:50
    rhoK=vector*tvec(i)+[rho0;K0];
    X=vpasolve(subs([eqn1;eqn2;eqn3],[rho,K],[rhoK(1),rhoK(2)]));
    Bvec=[Bvec,X.B];
end

Bvec3=[];
for i=51:100
    rhoK=vector*tvec(i)+[rho0;K0];
    X=vpasolve(subs([eqn1;eqn2;eqn3],[rho,K],[rhoK(1),rhoK(2)]));
    Bvec3=[Bvec3,X.B];
end

T=[tvec(1:50),tvec(51:100),tvec(51:100),tvec(51:100)];
Bt=[Bvec(1:50),Bvec3(1,:),Bvec3(2,:),Bvec3(3,:)];
```

```
figure(2)
plot(T,Bt,'.')
xlabel('T')
ylabel('B')
```

Figure 8

```
s=.02;
S=20;
c=200;
m=2;
b=3;
r=5;
a=1000;

syms R L B rho K
assume([R L B rho K], 'Real')
assume([R L B rho K], 'Positive')

eqnB = @(K, rho, B) s + (S-s)*K^b/(K^b+B^b) - B*(1+a*c/((m^r*rho^r*(1+B)^r+1)*(1+c*B)));

fimplicit3(eqnB)
xlabel('K');
ylabel('rho');
zlabel('B');
```

```
set(gca, 'Xscale','log')

set(gca, 'Yscale','log')

zlim([0,10])
```

Supplementary Materials: Figure 12

```
syms x y a m

assume([x y a m],'Real')

assume([x y a m],'Positive')


figure

fimplicit3(@(a,m,x) (a/(1+(2/(1+x^m))^m)) - x, [0 3 0 3 0 3])

xlabel('\alpha_1'); ylabel('m'); zlabel('x');

title('2-gene network, plot x vary \alpha_1,m')


figure

fimplicit3(@(a,m,y) (2/(1+(a/(1+y^m))^m)) - y, [0 3 0 3 0 3])

xlabel('\alpha_2'); ylabel('m'); zlabel('y');

title('2-gene network, plot y vary \alpha_2,m')
```

Supplementary Materials: Figure 13

```
alpha=2;

x = -1:.2:3;

y = -1:.2:3;

[X,Y] = meshgrid(x,y);
```

```
m = 10; %vary m

V1 = alpha./(1+Y.^m)-X

V2 = alpha./(1+X.^m)-Y

uV1=V1./sqrt(V1.^2+V2.^2);

uV2=V2./sqrt(V1.^2+V2.^2);

hold on

quiver(X,Y,uV1,uV2,.5,'c')

plot(1,1,"*r",2, 0,"ro",0, 2,"ro",x,x,'r')

str={'saddle',{'sink','high CDK2','low Rb'},{'sink','low CDK2','high Rb'}}

text([1.1 2.1 0.1],[1 0 2],str)

text(-.5,-.5,'x=y','Color','red')

xlabel('x')

ylabel('y')

title('2-gene vector field, m=10, \alpha=2')

hold off
```

Supplementary Materials: Figures 14 - 16

```
alpha=2;

m=3;


%% Solution curves and eigenspace


figure(1)

vec = linspace(0,4,3);
```

```
plot3(1,1,1,'k*')
hold on


syms x y z


%eigenvectors of the saddle
x0=[1,1,1] % saddle pt
DxV=jacobian([
    (alpha/(1+z^m))-x;
    ((alpha*x^m)/(1+x^m))-y;
    (alpha/(1+y^m))-z],[x,y,z]);
A=double(subs(DxV,[x,y,z,alpha,m],[x0,alpha,m]));
[evec,eval]=eig(A);
real1=(evec(:,1)+evec(:,2))/2;
real2=A*real1;
%eigenspace
xs=linspace(0,2,5);
ys=linspace(0,2,5);
[Xs,Ys]=meshgrid(xs,ys);


a=((real1/norm(real1))'+x0)-x0;
b=((real2/norm(real2))'+x0)-x0;
n=cross(a,b)
Z=(-n(1).*(Xs-1)-n(2).*(Ys-1))./n(3) + 1;
```

```
surf(Xs,Ys,Z,'FaceColor',[1 0 0],'EdgeColor','none','FaceAlpha',.4)

eigdir=[((real1/norm(real1))'+x0);x0;((real2/norm(real2))'+x0)];

plot3(eigdir(:,1),eigdir(:,2),eigdir(:,3),'m-','LineWidth',2)


clear x y z

t=[0,20];

for i=1:length(vec)

    for j=1:length(vec)

        for k=1:length(vec)

            X0=[vec(i),vec(j),vec(k)];

            [t,x_out]=ode45(@xdot, t, X0);

            plot3(x_out(:,1),x_out(:,2),x_out(:,3),'b:')

            plot3(x_out(end,1),x_out(end,2),x_out(end,3),'k*')

        end

    end

end


[t,x_out]=ode45(@xdot, [0 100], [0.5 0.5 2.5]);

sink1=x_out(end,:);

[t,x_out]=ode45(@xdot, [0 100], [2.5,2.5,.5]);

sink2=x_out(end,:)-[0,0,.05];


grid on
```

```
xlim([0 2])
ylim([0 2])
zlim([0 2])
xlabel('x')
ylabel('y')
zlabel('z')
title('Plot of solution curves divided by the eigenspace')
legend('Equilibria','Eigenspace')
hold off

%% Surfaces

[x,y] = meshgrid(linspace(-1,10));

yy =  (2*x.^m)./(x.^m+1);
zz =  2./(x.^m+1);
xx =  2./(x.^m+1);

figure(2)
plot3(1,1,1,'r*','MarkerSize',12)
hold on
surf(xx,y,x,'FaceAlpha',0.2,'FaceColor','b','LineStyle','none')
surf(x,yy,y,'FaceAlpha',0.2,'FaceColor','r','LineStyle','none')
surf(x,y,zz,'FaceAlpha',0.2,'FaceColor','g','LineStyle','none')
```

```
plot3(sink1(1),sink1(2),sink1(3),'ko','MarkerSize',12)
plot3(sink2(1),sink2(2),sink2(3),'ko','MarkerSize',12)
%surf(x,yy,xx)
xlim([0 2])
ylim([0 2])
zlim([0 2])
xlabel('x')
ylabel('y')
zlabel('z')
grid on
legend('saddle','x','y','z','sink')
title('3-gene solution surfaces and nullclines')
hold off

%% Vector plot
clear x y z
figure(3)
x = 0:.5:2.5;
y = 0:.5:2.5;
z = 0:.5:3.5;
[X,Y,Z] = meshgrid(x,y,z);
V1 = alpha./(1+Z.^m)-X;
V2 = alpha*X.^m./(1+X.^m)-Y;
```

```
V3 = alpha./(1+Y.^m)-Z;

uV1=V1./sqrt(V1.^2+V2.^2+V3.^2);

uV2=V2./sqrt(V1.^2+V2.^2+V3.^2);

uV3=V3./sqrt(V1.^2+V2.^2+V3.^2);

quiver3(X,Y,Z,uV1,uV2,uV3,.3)

hold on

plot3(1,1,1,'r*')

plot3(sink1(1),sink1(2),sink1(3),'ko')

plot3(sink2(1),sink2(2),sink2(3),'ko')

xlim([0 2])

ylim([0 2])

zlim([0 2])

xlabel('x')

ylabel('y')

zlabel('z')

title('3-gene vector field, m=3, \alpha=2')

hold off


%% Projections

clear x y z X Y Z

x = linspace(0,4,20);

y = linspace(0,4,20);

z = linspace(0,4,20);

Y =  (2*x.^m)./(x.^m+1);
```

```
Z =  2./(y.^m+1);
X =  2./(z.^m+1);

figure(4)
% XY
nexttile
plot(x,Y)
title('Projection on x-y plane')
xlabel('x')
ylabel('y')
% ZX
nexttile
plot(X,z)
title('Projection on z-x plane')
xlabel('x')
ylabel('z')
% YZ
nexttile
plot(y,Z)
title('Projection on y-z plane')
xlabel('y')
ylabel('z')

%% ODE function
```

```
function dx=xdot(t,x)

alpha = 2;

m = 3;

dx=zeros(3,1);

dx(1)=(alpha/(1+x(3)^m))-x(1);

dx(2)=((alpha*x(1)^m)/(1+x(1)^m))-x(2);

dx(3)=(alpha/(1+x(2)^m))-x(3);

end
```

References

[1] Jiyoung Lee et al. Network of mutually repressive metastasis regulators can promote cell heterogeneity and metastatic transition. *PNAS*, vol. 111,3 (2014): E364-73. doi:10.1073/pnas.1304840111.

[2] Sabrina L. Spencer et al. The proliferation-quiescence decision is controlled by a bifurcation in cdk2 activity at mitotic exit. *Cell*, 155(2):369–383, 2013.

[3] Willy J.F Govaerts. *Numerical Methods for bifurcations of dynamical equilibrium.* SIAM, 2000.

[4] John Guckenheimer and Philip Holmes. *Non-linear Oscillations, Dynamical Systems and Bifurcation of Vector Fields.* Springer, 1983.

[5] John Guckenheimer and Yuri A. Kuznetsov. Cusp bifurcation. `http://www.scholarpedia.org/article/Cusp_bifurcation`, 2013.

[6] Pierre Gönczy and Yemima Budirahardja. Coupling the cell cycle to development. *Development*, vol. 136,17: 2861-72., 2009.

[7] Marc W Kirschner and Li Victor C. Molecular ties between the cell cycle and differentiation in embryonic stem cells. *Proceedings of the National Academy of Sciences of the United States of America*, 111,26: 9503-8, 2014.

[8] Yuri Kuznetsov. *Elements of applied Bifurcation Theory, 3rd edition.* Springer, 2004.

[9] Yuri A. Kuznetsov. Saddle-node bifurcation. `http://www.scholarpedia.org/article/Saddle-node_bifurcation`, 2015.

[10] Indika Rajapakse and Steven Smale. Mathematics of the genome. *Foundations of Computational mathematics*, 17;1195-1217, 2017.

[11] Indika Rajapakse and Steven Smale. The pitchfork bifurcation. *International Journal of Bifur-*

cation and Chaos, 27.09:1750132, 2017.
